# Supplementary material for: Modeling nonlinear oscillator networks using physics-informed hybrid reservoir computing
Source: Sci Rep. 2025 Jul 2;15:22497. doi: 10.1038/s41598-025-03957-x (PMC12219108; doi:10.1038/s41598-025-03957-x)
Supplement: Supplementary file 1 — Supplementary Information. [file 41598_2025_3957_MOESM1_ESM.pdf]

Supplementary Information

Modeling Nonlinear Oscillator Networks Using Physics-Informed Hybrid Reservoir Computing

Andrew Shannon, Conor Houghton, David Barton, Martin Homer

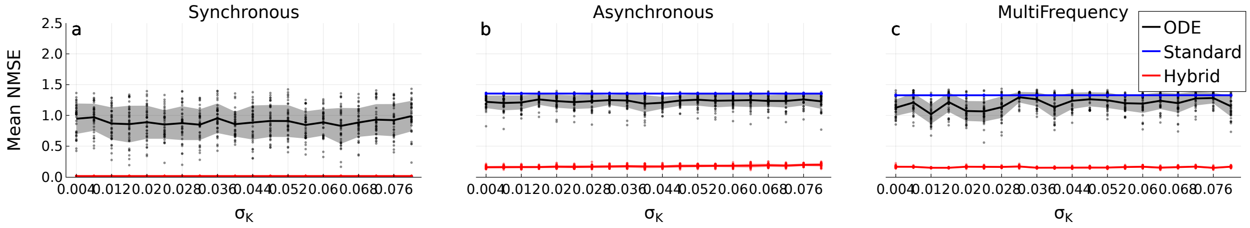

Supplementary Figure S1. Parameter error task parameter sweeps evaluating the hybrid RC's prediction of NLON trajectories with parameter error in its expert model as the standard deviation of the coupling strength error, σ_K, is varied. Mean NMSE in the prediction of the hybrid RC (red), standard RC (blue), and the base ODE model (black) across the three dynamical regimes. Column - dynamical regime: Synchronous (**a**), Asynchronous (**b**), Multi-Frequency (**c**). Individual dots are individual reservoir/ODE instantiations (40), each representing the mean NMSE across 60 forecasts, (20 for each realization of a ground truth regime). Solid lines are the mean NMSE across the reservoir/ODE instantiations. Shaded regions are one standard deviation across reservoir/ODE instantiations.

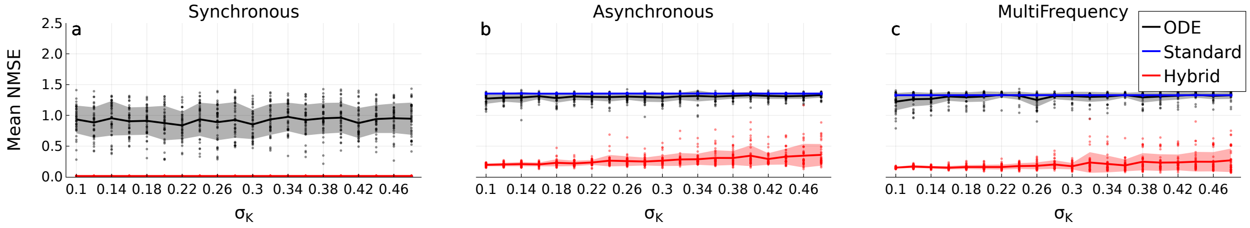

Supplementary Figure S2. Parameter error task parameter sweeps evaluating the hybrid RC's prediction of NLON trajectories with parameter error in its expert model as the standard deviation of the coupling strength error, σ_K,is varied across high values. Mean NMSE in the prediction of the hybrid RC (red), standard RC (blue), and the base ODE model (black) across the three dynamical regimes. Column - dynamical regime: Synchronous (**a**), Asynchronous (**b**), Multi-Frequency (**c**). Individual dots are individual reservoir/ODE instantiations (40), each representing the mean NMSE across 60 forecasts, (20 for each realization of a ground truth regime). Solid lines are the mean NMSE across the reservoir/ODE instantiations. Shaded regions are one standard deviation across reservoir/ODE instantiations.

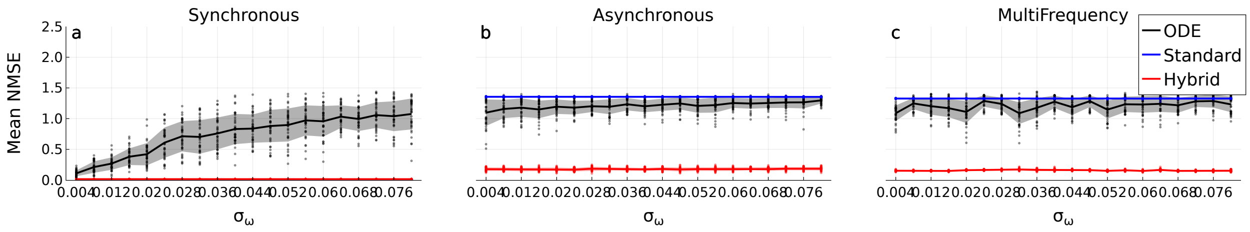

Supplementary Figure S3. Parameter error task parameter sweeps evaluating the hybrid RC's prediction of NLON trajectories with parameter error in its expert model as the standard deviation of the natural frequency error, σ_ω, is varied. Mean NMSE in the prediction of the hybrid RC (red), standard RC (blue), and the base ODE model (black) across the three dynamical regimes. Column - dynamical regime: Synchronous (**a**), Asynchronous (**b**), Multi-Frequency (**c**). Individual dots are individual reservoir/ODE instantiations (40), each representing the mean NMSE across 60 forecasts, (20 for each realization of a ground truth regime). Solid lines are the mean NMSE across the reservoir/ODE instantiations. Shaded regions are one standard deviation across reservoir/ODE instantiations.

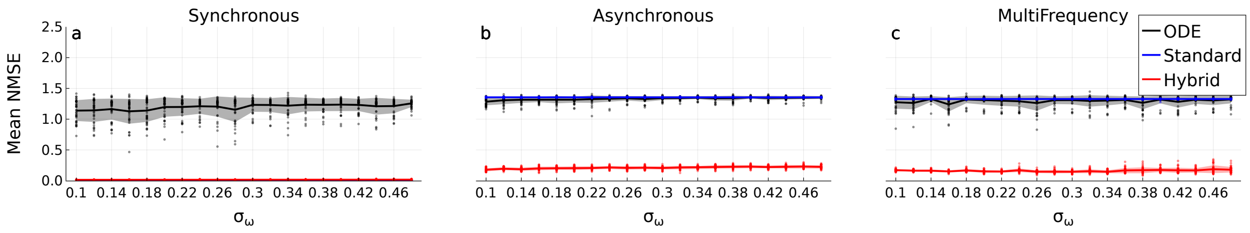

Supplementary Figure S4. Parameter error task parameter sweeps evaluating the hybrid RC's prediction of NLON trajectories with parameter error in its expert model as the standard deviation of the natural frequency error, σ_ω, is varied across high values. Mean NMSE in the prediction of the hybrid RC (red), standard RC (blue), and the base ODE model (black) across the three dynamical regimes. Column - dynamical regime: Synchronous (**a**), Asynchronous (**b**), Multi-Frequency (**c**). Individual dots are individual reservoir/ODE instantiations (40), each representing the mean NMSE across 60 forecasts, (20 for each realization of a ground truth regime). Solid lines are the mean NMSE across the reservoir/ODE instantiations. Shaded regions are one standard deviation across reservoir/ODE instantiations.

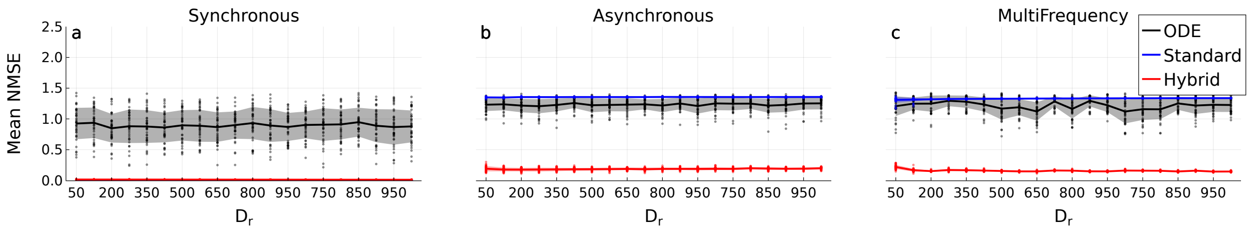

Supplementary Figure S5. Parameter error task parameter sweeps evaluating the hybrid RC's prediction of NLON trajectories with parameter error in its expert model as the reservoir size is varied. Mean NMSE in the prediction of the hybrid RC (red), standard RC (blue), and the base ODE model (black) across the three dynamical regimes. Column - dynamical regime: Synchronous (**a**), Asynchronous (**b**), Multi-Frequency (**c**). Individual dots are individual reservoir/ODE instantiations (40), each representing the mean NMSE across 60 forecasts, (20 for each realization of a ground truth regime). Solid lines are the mean NMSE across the reservoir/ODE instantiations. Shaded regions are one standard deviation across reservoir/ODE instantiations.

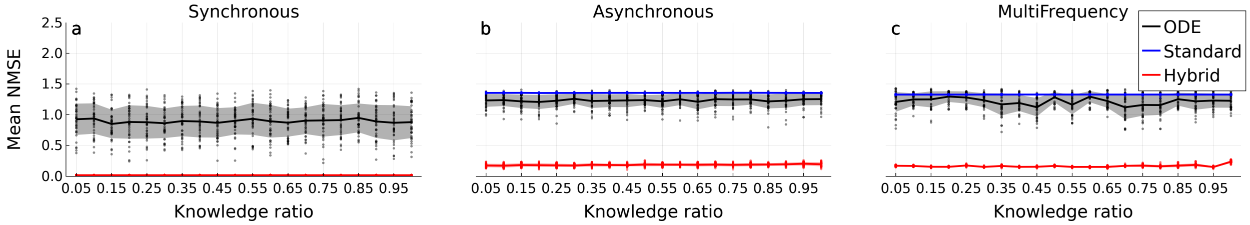

Supplementary Figure S6. Parameter error task parameter sweeps evaluating the hybrid RC's prediction of NLON trajectories with parameter error in its expert model as the knowledge ratio is varied. Mean NMSE in the prediction of the hybrid RC (red), standard RC (blue), and the base ODE model (black) across the three dynamical regimes. Column - dynamical regime: Synchronous (**a**), Asynchronous (**b**), Multi-Frequency (**c**). Individual dots are individual reservoir/ODE instantiations (40), each representing the mean NMSE across 60 forecasts, (20 for each realization of a ground truth regime). Solid lines are the mean NMSE across the reservoir/ODE instantiations. Shaded regions are one standard deviation across reservoir/ODE instantiations.

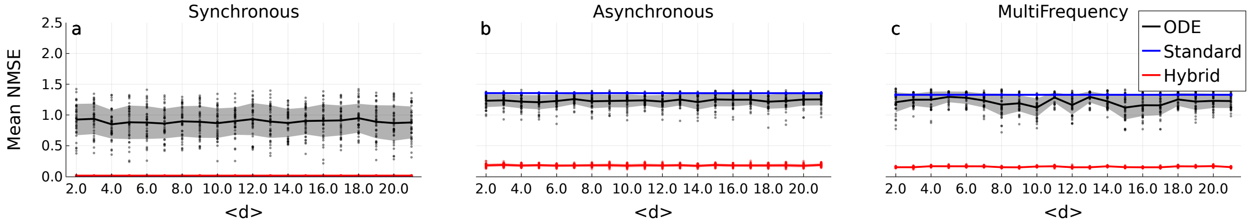

Supplementary Figure S7. Parameter error task parameter sweeps evaluating the hybrid RC's prediction of NLON trajectories with parameter error in its expert model as the mean degree is varied. Mean NMSE in the prediction of the hybrid RC (red), standard RC (blue), and the base ODE model (black) across the three dynamical regimes. Column - dynamical regime: Synchronous (**a**), Asynchronous (**b**), Multi-Frequency (**c**). Individual dots are individual reservoir/ODE instantiations (40), each representing the mean NMSE across 60 forecasts, (20 for each realization of a ground truth regime). Solid lines are the mean NMSE across the reservoir/ODE instantiations. Shaded regions are one standard deviation across reservoir/ODE instantiations.

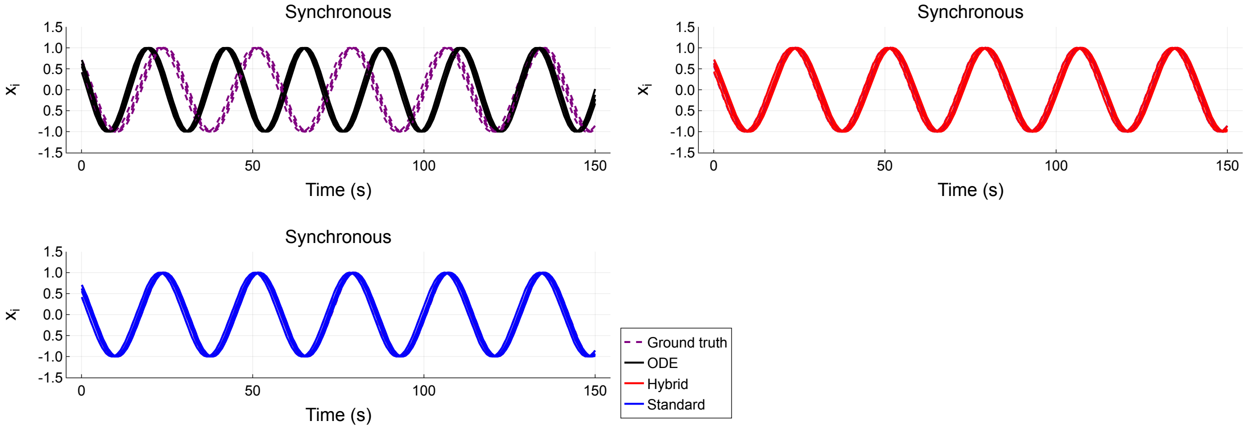

Supplementary Figure S8. Example synchronous regime phase-component trajectories forecast by each model compared to the ground truth from the parameter error task's standard Kuramoto model. Base ODE (black), standard RC (blue), hybrid RC (red), ground truth (dashed purple). Both the standard and hybrid RCs accurately forecast the synchronous dynamics. The ODE does produce synchronous dynamics, but the error in its natural frequencies causes the forecast to drift. Parameters from the tenth index of the second σ_K sweep corresponding to $\sigma_K = 0.28$, all other parameters at baseline.

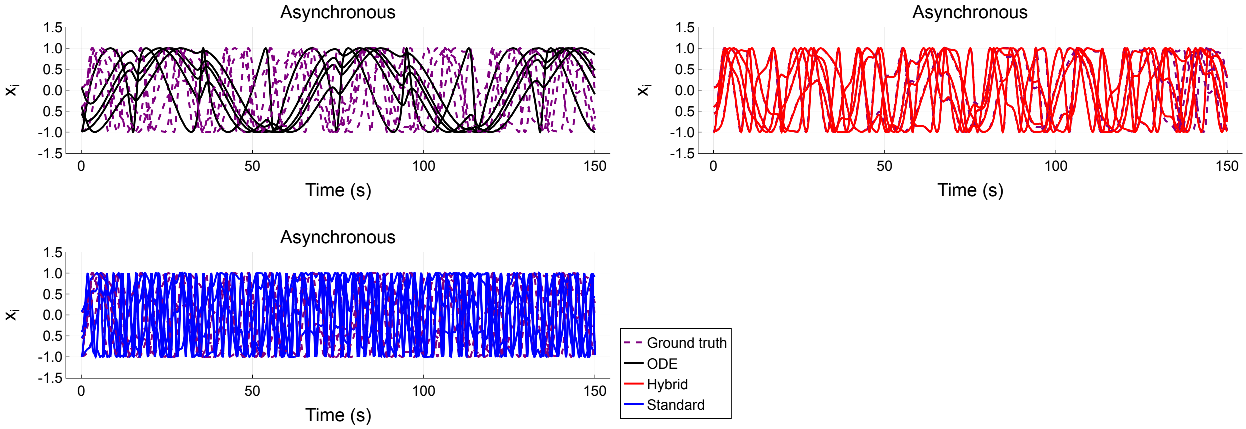

Supplementary Figure S9. Example asynchronous regime phase-component trajectories forecast by each model compared to the ground truth from the parameter error task's standard Kuramoto model. Base ODE (black), standard RC (blue), hybrid RC (red), ground truth (dashed purple). The error in both the coupling strength and natural frequencies of the ODE model is enough to stop it accurately forecasting the complex asynchronous dynamics. The hybrid RC does exceptionally well for this particular instantiation and parameter setting. In contrast, the standard RC does not. Parameters from the tenth index of the second σ_K sweep corresponding to $\sigma_K = 0.28$, all other parameters at baseline.

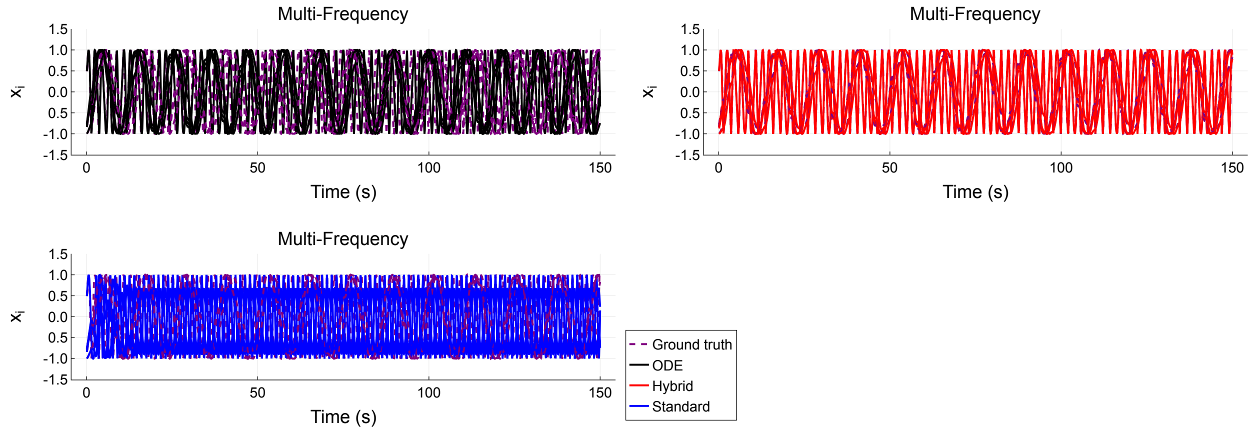

Supplementary Figure S10. Example multi-frequency regime phase-component trajectories forecast by each model compared to the ground truth from the parameter error task's standard Kuramoto model. Base ODE (black), standard RC (blue), hybrid RC (red), ground truth (dashed purple). As for the synchronous regime, the ODE model correctly captures the synchronous low frequency cluster's and phase-locked high frequency oscillator's behavior but drifts away due to the natural frequency error. The hybrid RC nearly perfectly forecasts the dynamics. The standard RC struggles with predicting the high frequency oscillator, in contrast to its excellent performance on the synchronous regime; the strong time-scale separation may be the cause of this. Parameters from the tenth index of the second σ_K sweep corresponding to $\sigma_K = 0.28$, all other parameters at baseline.

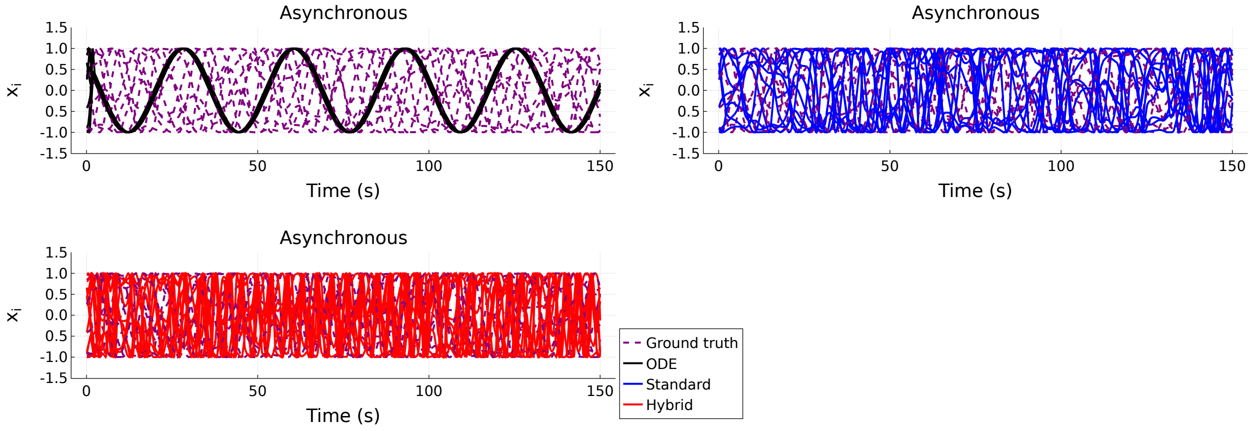

Supplementary Figure S11. Example asynchronous regime phase-component trajectories forecast by each model compared to the ground truth from the residual physics task's bi-harmonic Kuramoto model. Base ODE (black), standard RC (blue), hybrid RC (red), ground truth (dashed purple). The base ODE model forecast achieves a higher valid time than the hybrid and standard RC but is clearly failing to capture the main feature of the dynamics once this time has elapsed as it synchronizes. The reservoirs can produce more realistic oscillations, with the standard reservoir better capturing the qualitative dynamics for this parameter set. Parameters from the third index of the spectral radius sweep corresponding to a spectral radius of 0.3, all other parameters at baseline.

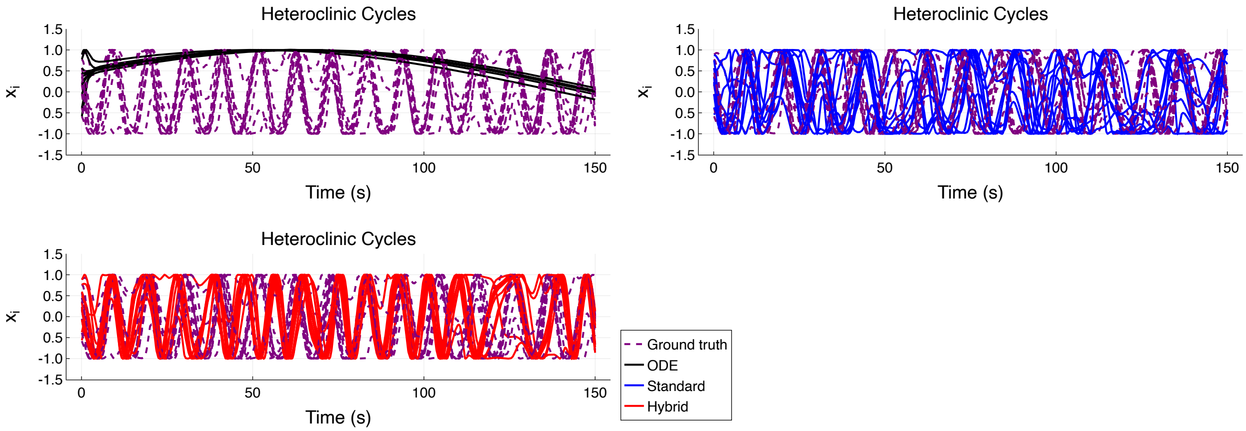

Supplementary Figure S12. Example heteroclinic cycles regime phase-component trajectories forecast by each model compared to the ground truth from the residual physics task's bi-harmonic Kuramoto model. Base ODE (black), standard RC (blue), hybrid RC (red), ground truth (dashed purple). The base ODE model predicts slow synchronous trajectories, whilst the standard and hybrid RC better recreate the gross features of the dynamics. The hybrid RC in particular seems to best capture the underlying oscillation frequency and also has some oscillators departing from and returning to the main attracting cluster as is required for this regime. Parameters from the first index of the input scaling sweep corresponding to an input scaling of 0.05, all other parameters at baseline.

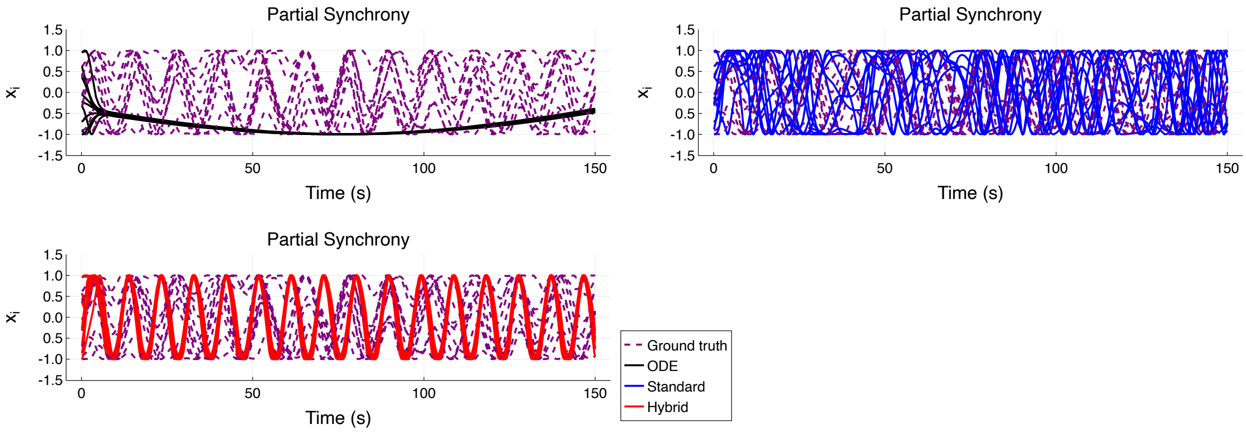

Supplementary Figure S13. Example partial synchrony regime phase-component trajectories forecast by each model compared to the ground truth from the residual physics task's bi-harmonic Kuramoto model. Base ODE (black), standard RC (blue), hybrid RC (red), ground truth (dashed purple). The base ODE model fails to capture the partial synchrony regime, predicting slow, synchronous dynamics. In contrast the hybrid RC successfully captures the underlying oscillation frequency. The hybrid RC forecasts overly synchronous trajectories however, failing to capture the partially synchronous, distributional behavior characteristic of this regime. Parameters from the first index of the input scaling sweep corresponding to an input scaling of 0.05, all other parameters at baseline.

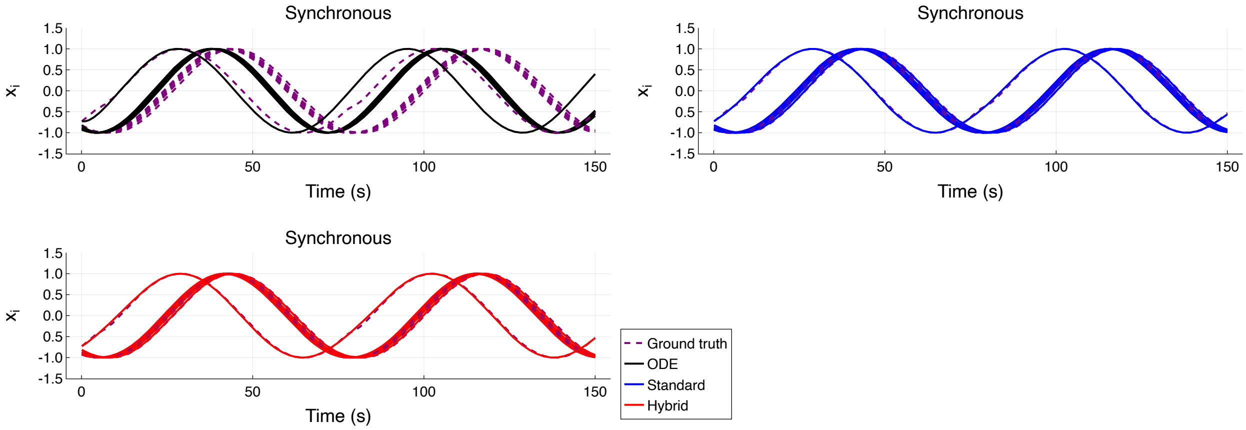

Supplementary Figure S14. Example synchronous regime phase-component trajectories forecast by each model compared to the ground truth from the residual physics task's bi-harmonic Kuramoto model. Base ODE (black), standard RC (blue), hybrid RC (red), ground truth (dashed purple). All three models do well on the synchronous regime, with the standard and hybrid RC often reaching the maximum 250 second valid time. The base ODE however drifts due to parameter error. Parameters from the first index of the input scaling sweep corresponding to an input scaling of 0.05, all other parameters at baseline.

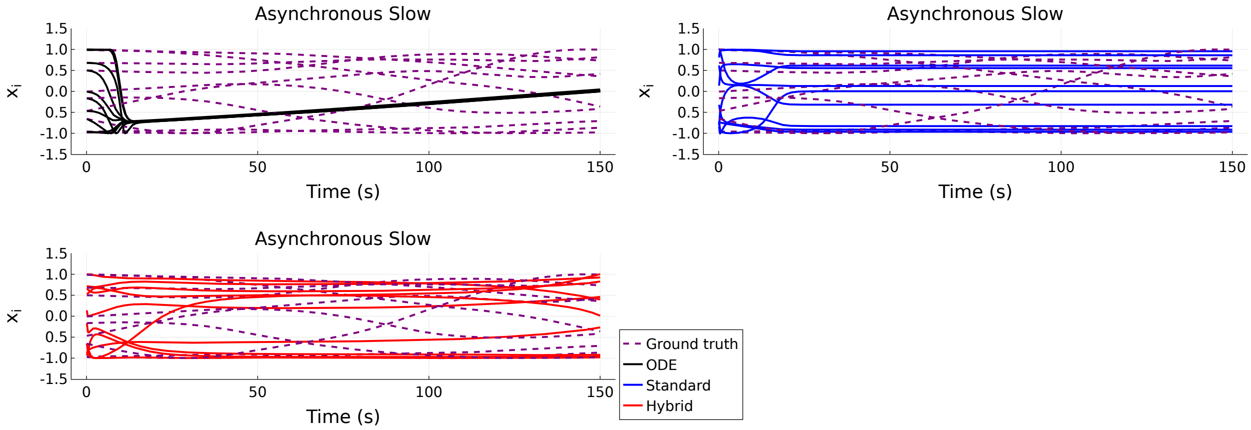

Supplementary Figure S15. Example slow asynchronous regime phase-component trajectories forecast by each model compared to the ground truth from the residual physics task's bi-harmonic Kuramoto model. Base ODE (black), standard RC (blue), hybrid RC (red), ground truth (dashed purple). The slow, synchronous base ODE model forecast achieves a higher valid time than the hybrid and standard RC but is clearly failing to capture the main feature of the dynamics once this time has elapsed. The standard RC also fails, but through what appears to be falling into a fixed point solution. Parameters from the first index of the input scaling sweep corresponding to an input scaling of 0.05, all other parameters at baseline.

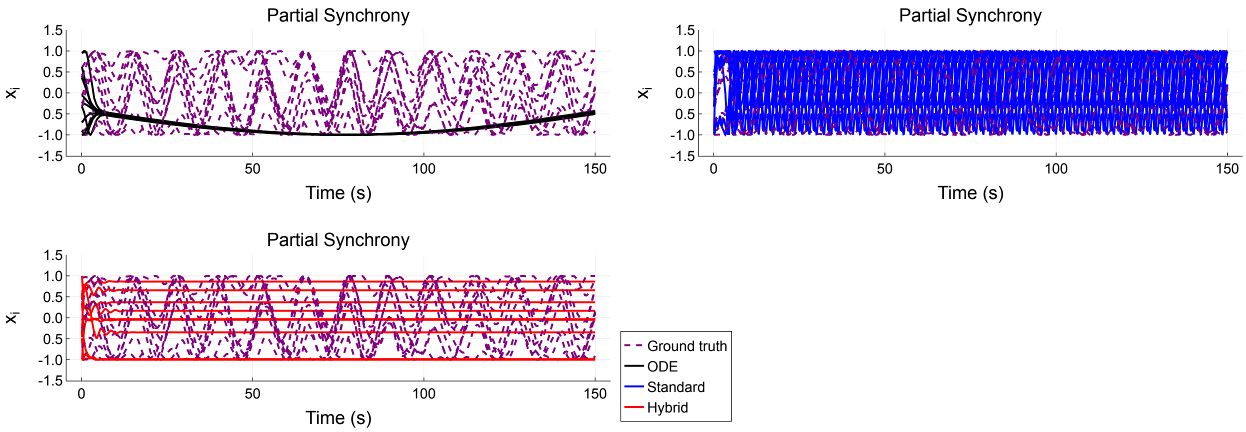

Supplementary Figure S16. Example failed partial synchrony regime phase-component trajectories forecast by each model compared to the ground truth from the residual physics task's bi-harmonic Kuramoto model. Base ODE (black), standard RC (blue), hybrid RC (red), ground truth (dashed purple). In this case (parameters at baseline with input scaling set to 1.9) the hybrid and standard RC's fail by producing steady state and high frequency trajectories respectively.

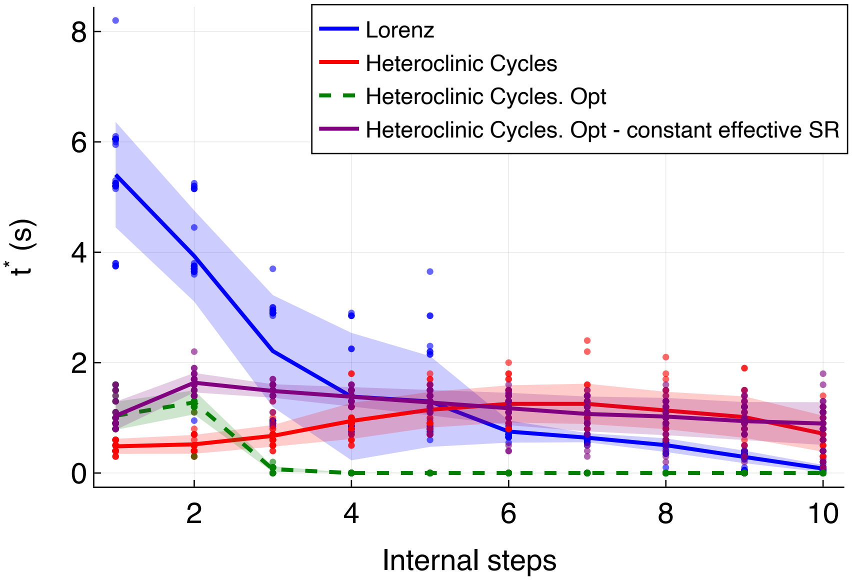

Supplementary Figure S17. Multi-step reservoir investigation. Mean valid times achieved by 30 multi-step reservoir instantiations as the number of internal steps varies on the Lorenz system (spectral radius 0.5, input scaling 0.15, regularization 0.000001, others at baseline)(blue), and the heteroclinic cycles regime under different parameter tunings. For un-tuned parameters (spectral radius 0.5, input scaling 0.15, regularization 0.000001, others at baseline)(red), seven internal steps is a local optimum, significantly more than the usual single step. When the parameters are tuned for one internal step however (spectral radius 0.05, input scaling 0.05, regularization 0.0001) (green) this optimum is only two steps. Subsequently, the performance drops off rapidly as the number of internal steps is increased. This may be due to the spectral radius being smaller in the optimized case than the untuned case such that the extra internal steps form an *effective spectral radius* that is too small. It is also possible that the representations produced by a single step of the reservoir are already rich enough for the dynamical systems considered. When the spectral radius is adjusted from the tuned value such that the *effective spectral radius*, defined as the spectral radius of $\mathbf{A}^m$ where $\mathbf{A}$ is the internal reservoir connectivity matrix, and m is the number of internal steps, is kept constant (at 0.05), the rapid decrease in valid time is ameliorated (purple). Maximum valid times achieved across these cases for the heteroclinic cycles regime do not differ greatly, suggesting optimization of the spectral radius (and other parameters, as conducted in this study) may be equivalent to optimizing the number of internal steps. Dots: individual reservoir instantiation valid time on single test span. Solid/Dashed lines: mean valid time across reservoirs. Shaded regions: one standard deviation across reservoirs.

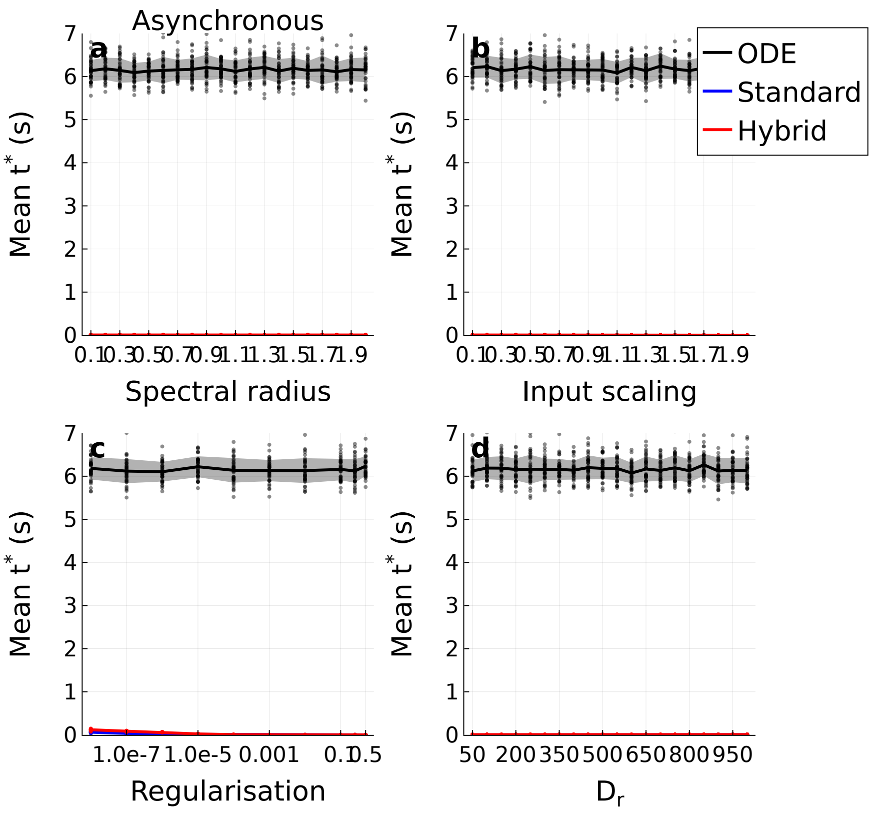

Supplementary Figure S18. Residual Physics task parameter sweeps evaluating the hybrid RC's prediction of NLON trajectories with missing dynamics in its expert model in a slow asynchronous regime. Mean valid time of the prediction of the hybrid RC (red), standard RC (blue), and the base ODE model (black) as four different parameters are varied. Left to right, top to bottom - parameter varied: Spectral radius (**a**), Input scaling (**b**), Regularization (**c**), Reservoir size D_r (**d**). Individual dots are individual reservoir/ODE instantiations (40), each representing the mean NMSE across 60 forecasts, (20 for each realization of a ground truth regime). Solid lines are the mean NMSE across the reservoir/ODE instantiations. Shaded regions are one standard deviation across reservoir/ODE instantiations.

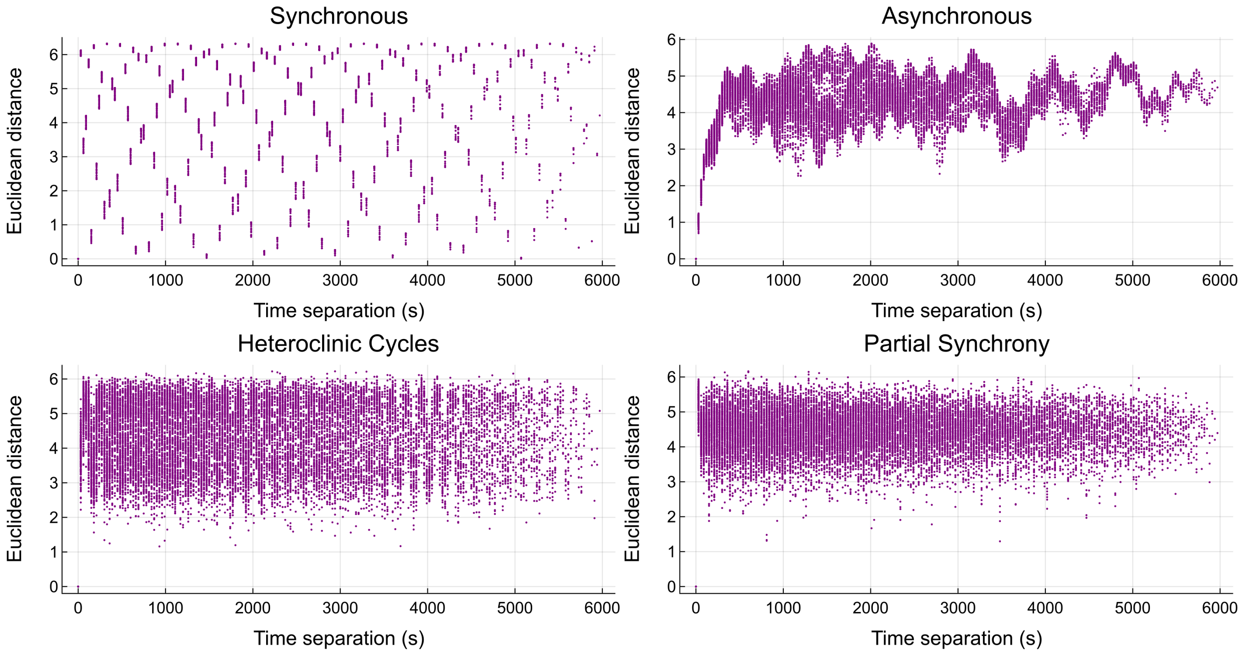

Supplementary Figure S19. Space-time separation plots of the ground truth trajectories of each dynamical regime of the bi-harmonic Kuramoto model from the residual physics task, with the slow asynchronous regime. These plot the pairwise separation between all pairs of points in the trajectory, with Euclidean distance on the y-axis and time on the x-axis. The slow asynchronous regime has a protracted region where for small time intervals, the Euclidean distance between points is close. This demonstrates how slow the trajectories are in comparison to the other regimes. We suggest that this is the cause of the training and prediction failures of both the standard and hybrid RCs on the slow asynchronous regime.

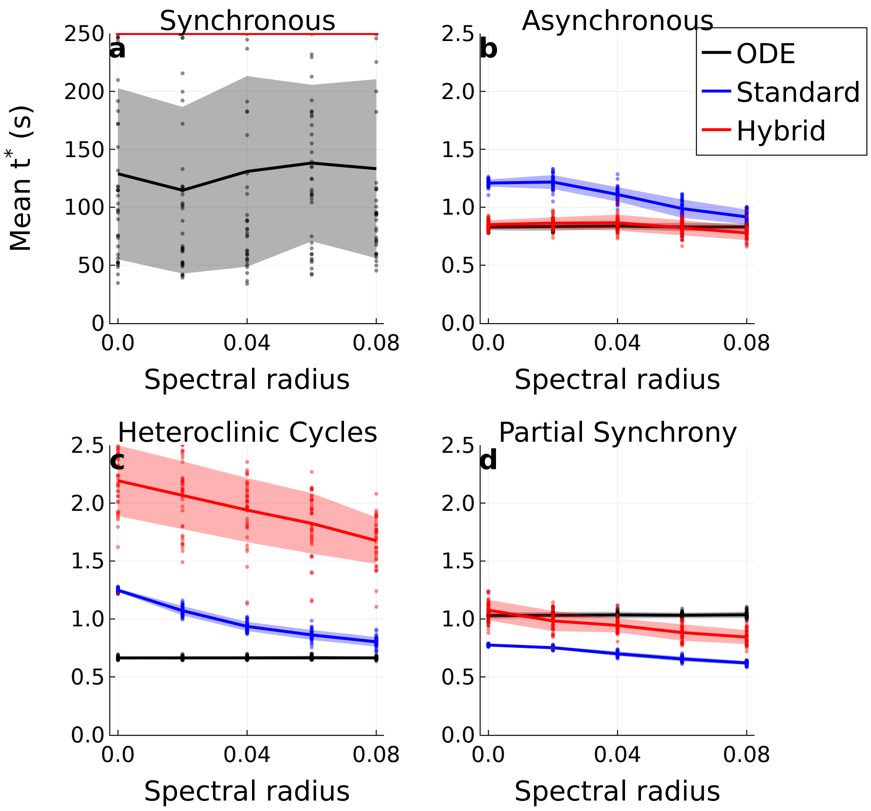

Supplementary Figure S20. Residual Physics task parameter sweeps evaluating the hybrid RC's prediction of NLON trajectories with missing dynamics in its expert model as the spectral radius of the reservoir is varied down to 0.0. Mean valid time of the prediction of the hybrid RC (red), standard RC (blue), and the base ODE model (black) across the four dynamical regimes. Left to right, top to bottom - dynamical regime: Synchronous (**a**), Asynchronous (**b**), Heteroclinic Cycles (**c**), Partial Synchrony (**d**). Individual dots are individual reservoir/ODE instantiations (40), each representing the mean NMSE across 60 forecasts, (20 for each realization of a ground truth regime). Solid lines are the mean NMSE across the reservoir/ODE instantiations. Shaded regions are one standard deviation across reservoir/ODE instantiations.

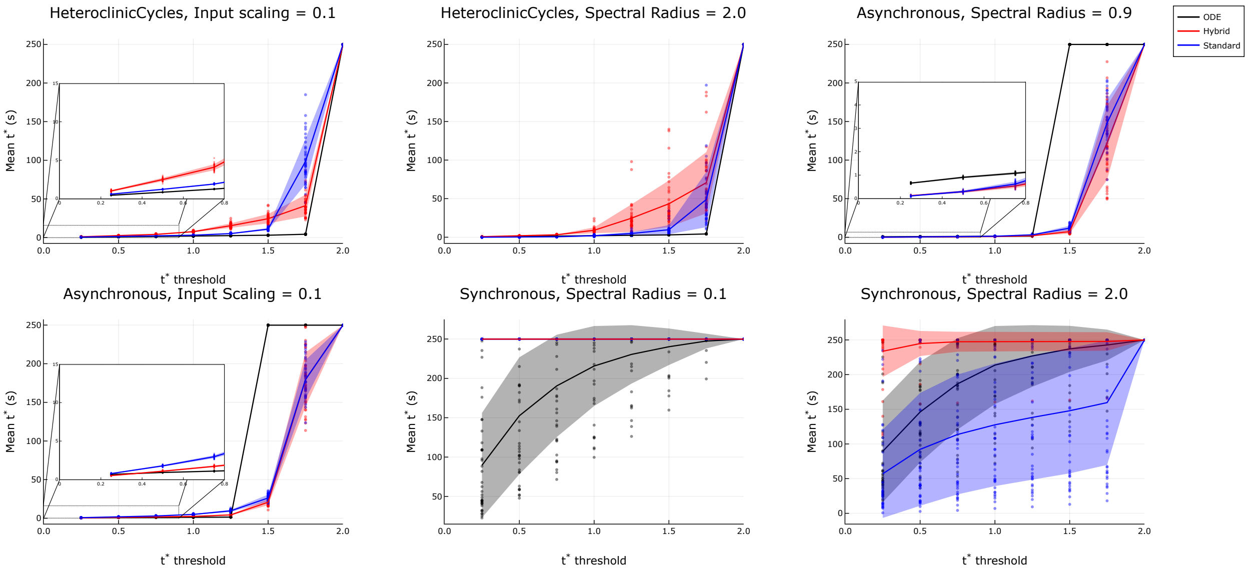

Supplementary Figure S21. Varying the threshold used in the valid time metric computation. Each subplot is at a particular parameter setting from the sweeps in Figure 9, for a given regime. Solid lines: mean mean valid time across tests and instantiations. Dots - mean valid time across tests per instantiation. Shaded region - one standard deviation over instantiations. A threshold of 2.0 is the maximum possible normalized mean square error, occurring when the ground truth state and model prediction are at opposite ends of the state space (20 dimensions). “Accurate” trajectories occur well below this, but this is subjective. A threshold of 0.4 is used in the paper, small variations about this do not significantly affect the ranking of the methods.

Code Implementation

Random Seeds The random number generator used to instantiate each reservoir and ODE parameter settings, as well as the parameter error sampling was of Mersenne Twister type, with a seed set to $1234 + parameter\_index$, where the $parameter\_index$ ranged from 1 to 20 where the parameter sweep was across 20 points.
